# R-spondin1 Controls Muscle Cell Fusion through Dual Regulation of Antagonistic Wnt Signaling Pathways

**DOI:** 10.1016/j.celrep.2017.02.036

**Published:** 2017-03-07

**Authors:** Floriane Lacour, Elsa Vezin, C. Florian Bentzinger, Marie-Claude Sincennes, Lorenzo Giordani, Arnaud Ferry, Robert Mitchell, Ketan Patel, Michael A. Rudnicki, Marie-Christine Chaboissier, Anne-Amandine Chassot, Fabien Le Grand

**Affiliations:** 1 Sorbonne Universités, UPMC Univ Paris 06, INSERM UMRS974, CNRS FRE3617, Center for Research in Myology, 75013 Paris, France; 2 Département de pharmacologie et physiologie, Faculté de médecine et des sciences de la santé, Université de Sherbrooke, Sherbrooke, J1H5N4 QC, Canada; 3 Sprott Center for Stem Cell Research, Ottawa Hospital Research Institute, Regenerative Medicine Program, Ottawa, K1H8L6 ON, Canada; 4 Department of Cellular and Molecular Medicine, Faculty of Medicine, University of Ottawa, Ottawa, K1H 8M5 ON, Canada; 5 School of Biological Sciences, University of Reading, RG6 6UB Reading, UK; 6 Freiburg Institute for Advanced Studies, Albert-Ludwigs-Universität Freiburg, 79104 Freiburg im Breisgau, Germany; 7 Université Côte d’Azur, INSERM, CNRS, iBV, 06108 Nice, France

**Keywords:** skeletal muscle, canonical Wnt signaling, non-canonical Wnt signaling, R-spondin, regeneration, muscle satellite cell

## Abstract

Wnt-mediated signals are involved in many important steps in mammalian regeneration. In multiple cell types, the R-spondin (Rspo) family of secreted proteins potently activates the canonical Wnt/β-catenin pathway. Here, we identify Rspo1 as a mediator of skeletal muscle tissue repair. First, we show that deletion of *Rspo1* results in global alteration of muscle regeneration kinetics following acute injury. We find that muscle progenitor cells lacking *Rspo1* show delayed differentiation due to reduced activation of Wnt/β-catenin target genes. Furthermore, muscle cells lacking *Rspo1* have a fusion phenotype leading to larger myotubes containing supernumerary nuclei both in vitro and in vivo. The increase in muscle fusion was dependent on downregulation of Wnt/β-catenin and upregulation of non-canonical Wnt7a/Fzd7/Rac1 signaling. We conclude that reciprocal control of antagonistic Wnt signaling pathways by Rspo1 in muscle stem cell progeny is a key step ensuring normal tissue architecture restoration following acute damage.

## Highlights

• Rspo1 controls muscle regeneration
• Absence of Rspo1 lowers Wnt/β-catenin signaling in muscle progenitor cells
• Rspo1 limits cell fusion in vitro and in vivo
• Non-canonical Wnt7a/Fzd7/Rac1 signaling is augmented in Rspo1-null cells

## Graphical Abstract

**Figure:**
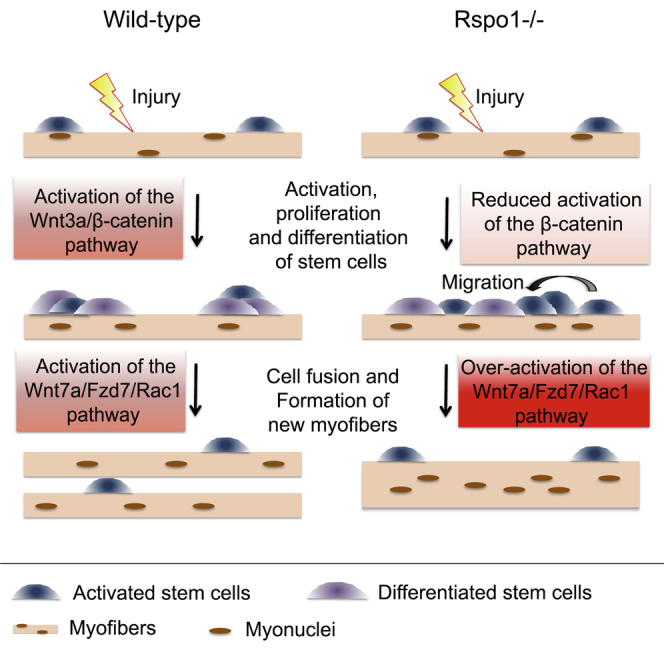

## Teaser

Using mice lacking the Wnt enhancer Rspo1, Lacour et al. demonstrate that Rspo1 controls muscle cell fusion during skeletal muscle regeneration. Rspo1-null muscle progenitor cells differentiate less efficiently but fuse extensively compared to wild-type cells. Rspo1 is shown to regulate the antagonism between canonical and non-canonical Wnt signaling pathways.

## Introduction

Adult muscle stem cells, called satellite cells (MuSCs), located around the differentiated myofibers exist in a quiescent state and are readily identified through the expression of the paired-box transcription factor Pax7 (Seale et al., 2000). Following injury to the host myofibers, they become activated and proliferate to give rise to muscle progenitor cells expressing the myogenic regulatory factors MyoD and Myogenin that act to promote differentiation (Chargé and Rudnicki, 2004). Differentiating myocytes will fuse together to form myotubes that eventually mature into new myofibers. Myocyte differentiation and fusion processes are highly regulated (Abmayr and Pavlath, 2012). Abnormal fusion can lead to the formation of giant, albeit disorganized, myofibers (Charrin et al., 2013) while a reduction in myocyte fusion potentially results in smaller muscles with myofibers containing fewer nuclei (Horsley et al., 2003). This is exemplified in skeletal muscle pathologies such as Duchenne muscle dystrophy (DMD). In dystrophic muscles, the continuous regeneration process leads to the formation of abnormal fibers, termed branched or split fibers, which are more prone to damage during contraction (Chan et al., 2007), lack force generation, and contribute to the disease progression (Head, 2010).

The Wnt signaling pathways are crucial regulators of myogenesis (von Maltzahn et al., 2012). The canonical Wnt/β-catenin pathway is required for muscle progenitor cell differentiation and plays a key role in skeletal muscle development (Borello et al., 2006, Anakwe et al., 2003) and repair following injury (Brack et al., 2009, Parisi et al., 2015). The non-canonical Wnt7a/Fzd7 signaling pathway plays a central role in three other aspects of muscle regeneration, namely, promoting the symmetric expansion of satellite stem cells through the planar cell polarity (PCP) pathway (Le Grand et al., 2009), enhancing MuSCs and myoblast migration (Bentzinger et al., 2014), and activating the anabolic Akt growth pathway in myofibers (von Maltzahn et al., 2011).

In many tissues, the activation of both canonical (Carmon et al., 2011) and non-canonical (Glinka et al., 2011) Wnt pathways is regulated by R-spondins. The R-spondin family of secreted proteins is comprised of four members (RSPO1–RSPO4) (de Lau et al., 2012). Each R-spondin has its own expression pattern and properties. R-spondins interact with leucine-rich repeat-containing G protein-coupled receptors (*LGR*) (de Lau et al., 2011) and potentiate Wnt signaling pathways by neutralizing the E3 ligases RNF43 and ZNRF3 that normally act to remove Wnt receptors from the surfaces of stem cells (Koo et al., 2012, Hao et al., 2012).

A number of studies reported the essential roles played by R-spondins during both embryonic development and adult tissue homeostasis or repair. For example, *Rspo1* is required for female sexual development and *Rspo1-null* mice show masculinized gonads (Parma et al., 2006, Chassot et al., 2008). In adult tissues, RSPO1 promotes growth of the intestinal epithelium (Kim et al., 2005), is a pancreatic beta-cell growth factor (Wong et al., 2010), and drives liver stem cell expansion (Huch et al., 2013). To date, the role of R-spondins in regenerative myogenesis remains unclear. *Rspo1*, *Rspo2*, and *Rspo3* were shown to be expressed by proliferating primary myoblasts, but only *Rpso1* and *Rspo3* were upregulated by the differentiation program (Han et al., 2011). A recent report documented that *Rspo1* transcription was strongly upregulated in primary myoblasts overexpressing Pax7 (Soleimani et al., 2012). We hence aimed to identify the function of *Rspo1* in steady-state muscle as well as during regeneration.

## Results

### Muscle Satellite Cells Express the Pax7 Target Gene *Rspo1*

**Figure 1:**
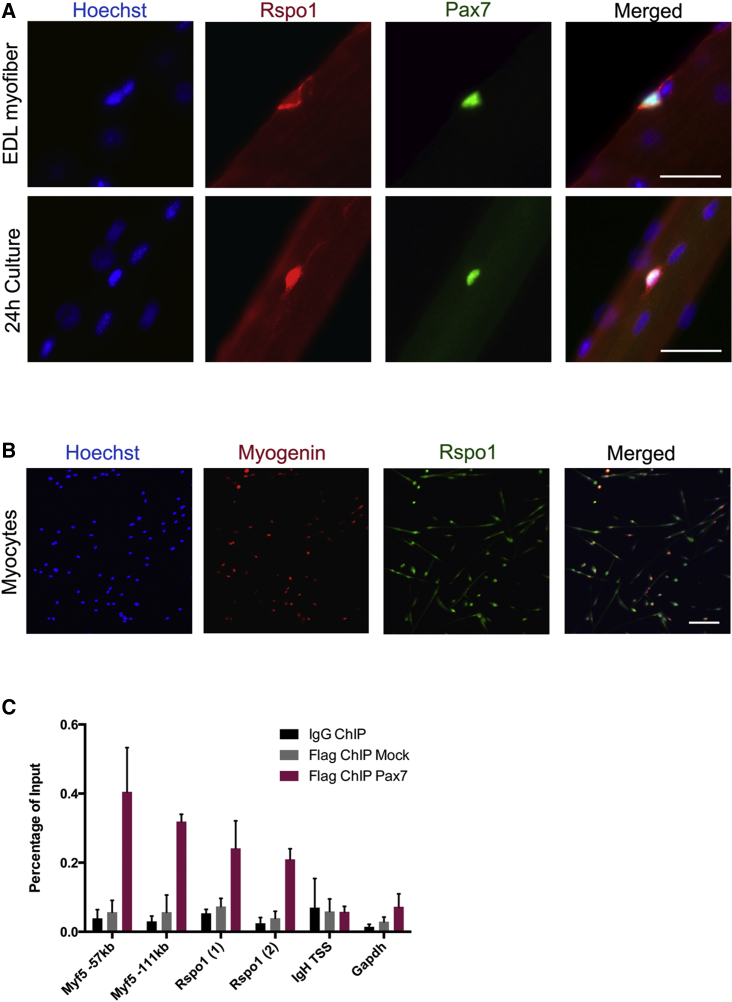
R-spondin1 Is Expressed in MuSCs and Primary Myocytes (A) RSPO1 (red) and PAX7 (green) immunolocalization in MuSCs on single myofibers. (B) MYOGENIN (red) and RSPO1 (green) immunolocalization in 24 hr-differentiating myocytes. (C) Occupancy of PAX7-Flag proteins at the promoters of *Myf5*, *Rspo1*, and *Gapdh* (control) genes in primary myoblasts. The input represents the relative enrichment of PAX7-Flag proteins compared to the controls (IgG and Flag-Mock). Rspo1 (1) and Rspo1 (2) indicate two different pairs of primers. Nuclei are stained with Hoechst (blue). Scale bars, 20 μm.

To profile Rspo1 expression in adult MuSCs, we performed immunocytochemical analysis for PAX7 and RSPO1 on myofibers isolated from extensor digitorum longus (EDL) muscles. We found that quiescent MuSCs expressed RSPO1 protein and that its expression increased following their activation (24 hr after isolation) (Figure 1A). Of note, RSPO1 was mostly cytoplasmic in quiescent MuSCs but appeared both in the cytoplasm and nucleus in activated MuSCs. In vitro analysis confirmed the expression of RSPO1 by differentiating primary muscle cells (Figure 1B).

We next tested if *Rspo1* is a direct target of Pax7 in adult myogenic cells. To this end, we determined, by chromatin immunoprecipitation (ChIP), whether PAX7 proteins interact with a putative binding site localized 35 kb upstream of *Rspo1* gene identified by ChIP coupled with deep sequencing (Soleimani et al., 2012). ChIP assays were performed using primary myogenic cell cultures expressing Pax7-Flag. They demonstrated that PAX7 proteins were bound to the *Rspo1* gene upstream regulatory region as well as to the positive controls (*Myf5* −111 kb and −57 kb enhancer regions), as relative to a mock IP and to the negative controls (Figure 1C) in muscle progenitor cells.

### *Rspo1* Is Not Required for Muscle Tissue Formation

We next investigated whether Rspo1 plays a role in the adult skeletal muscle tissue using *Rspo1* loss-of-function mice (Chassot et al., 2008). We did not observe any phenotypic difference in skeletal muscle histology (Figure S1A) and weights (Figure S1B) of young adult (8-week-old) *Rspo1-null* compared to control mice (controls are wild-type and *Rspo1*-*heterozygous* mice). In tibialis anterior (TA) muscles, loss of *Rspo1* expression did not alter the number of myofibers (Figure S1C), their size (Figure S1D), or cross-sectional area (CSA) (Figure S1E). In agreement with these findings, quantification of the number of myonuclei per myofiber (Figure S1F) and of the entire muscle CSA (Figure S1G) did not show any significant difference between the two genotypes. Furthermore, *Rspo1*-*null* and control animals showed similar number of Pax7-expressing MuSCs in cryosections of TA (Figure S1H), soleus, gastrocnemius, and triceps (data not shown). Immunocytochemical analysis for PAX7 and RSPO1 on *Rspo1-null* myofibers validated the specificity of the Rspo1 antibody (Figure S1I). These results show that *Rspo1* is not required for myofiber formation, cellular organization, nor for the establishment of the MuSC population.

### *Rspo1* Regulates Skeletal Muscle Tissue Regeneration

**Figure 2:**
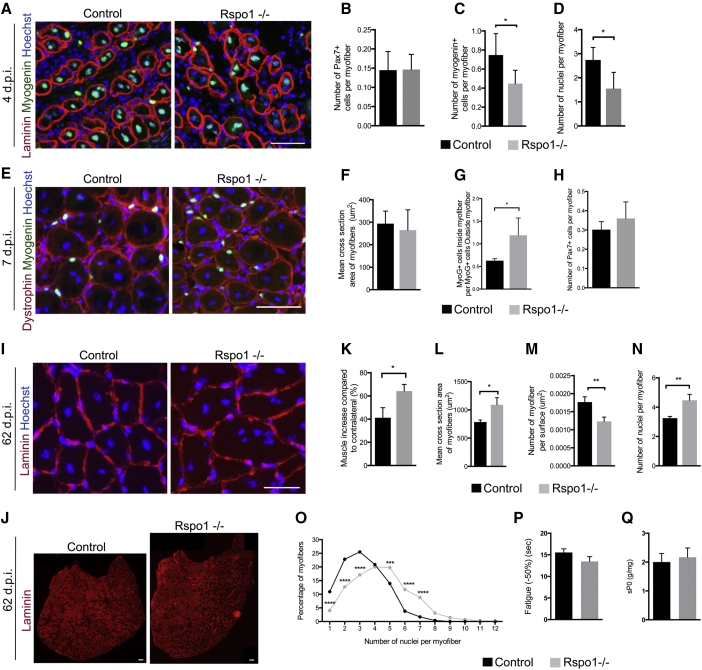
*Rspo1-null* Muscles Show Enhanced Regeneration Caused by a Delay of Muscle Progenitor Cell Differentiation and an Improved Fusion (A) LAMININ (red) and MYOGENIN (green) immunolocalization in TA muscles at 4 d.p.i. (B–D) Quantification of the number of PAX7-positive cells (B), MYOGENIN-positive cells (C), and myonuclei (D) per myofiber 4 d.p.i. (E) DYSTROPHIN (red) and MYOGENIN (green) immunolocalization at 7 d.p.i. (F) Mean CSA of myofibers in TA muscles at 7 d.p.i. (G) Quantification of the number of MYOGENIN-positive cells inside the myofibers showing a higher proportion of fused nuclei at 7 d.p.i. in *Rspo1-null* mice. (H) Quantification of the number of PAX7-positive cells per myofiber at 7 d.p.i. (I and J) LAMININ (red) immunolocalization on muscle sections at 62 d.p.i. (I) and a whole cross-section of regenerated 62 d.p.i. muscles (J). Images are virtual slides automatically assembled by the Axio Scan.Z1 microscope. (K and L) Quantification of TA muscle weights (K) and mean CSA (L) at 62 d.p.i. (M and N) Number of myofibers per surface unit (N) and of nuclei per myofiber (M) at 62 d.p.i. (O) Distribution of the percentage of myofibers depending on their nuclear number at 62 d.p.i. (P and Q) Fatigue resistance index (P) and specific maximal force (Q) of 62 d.p.i. muscles. Nuclei are stained with Hoechst (blue). Scale bars, 50 μm (A and E); 35 μm (I); 150 μm (J). Error bars indicate SD. ^∗^p value < 0.05; ^∗∗^p value < 0.01.

To investigate the role of *Rspo1* in skeletal muscle regeneration, we injured TA muscles of control and *Rspo1*-*null* mice with cardiotoxin injection. During the early phase of tissue regeneration at 4 days post-injury (d.p.i.) (Figure 2A), the number of Pax7^+^ progenitors was similar between control and *Rspo1*-*null* muscles (Figure 2B), but *Rspo1*-*null* muscles contained decreased numbers of Myogenin^+^-differentiating cells (Figure 2C) and of nuclei incorporated into myotubes (Figure 2D). This suggests a delay in the differentiation process. Later, at 7 d.p.i., (Figure 2E), newly formed myofibers were of a similar caliber in both genotypes (Figure 2F), but the number of Myogenin^+^ cells inside myofibers was increased in *Rspo1-null* muscles (Figure 2G), suggesting a compensatory enhancement of fusion in *Rspo1-null* muscles. At this time point, the number of Pax7^+^ progenitors remained similar between *Rspo1-null* and control muscles (Figure 2H). In this experimental setup, we did not observe any significant differences between male and female animals (Figure S2).

We next analyzed the morphology and cellular composition of fully regenerated muscles 62 d.p.i. (Figure 2I). Strikingly, regenerated *Rspo1*-null muscles were larger (Figures 2J) and heavier (Figure 2K) compared to control muscles. Regenerated *Rspo1-null* muscles were composed of larger myofibers (Figures 2L and 2M) containing a higher number of myonuclei (Figures 2N and 2O). Interestingly, quantification of sub-laminar MuSCs did not show any difference between both genotypes at 62 d.p.i.. To determine if improved muscle cell fusion resulted in functional changes, we compared the contractile properties of regenerated muscle in situ using an index of fatigue resistance (Figure 2P) and specific maximal force, a marker of force production capacity (Figure 2Q). These experiments indicate that regenerated Rspo1-null muscles, while composed of larger myofibers containing more myonuclei, are functional, because we found no reductions in the performed assays. Taken together, our results suggest that *Rspo1* is required for the proper timing of myogenic progenitor cells differentiation following injury and inhibits fusion in vivo.

### *Rspo1* Does Not Influence Primary Myoblasts Proliferation

To further analyze the behavior of muscle cells devoid of *Rspo1*, we cultured primary myoblasts expanded from MuSCs sorted by magnetic-activated cell sorting (MACS). *Rspo1*-*null* cells showed standard morphology and expressed the muscle progenitor marker M-CADHERIN (Figure S3A) (Cornelison and Wold, 1997). While proliferating, control and *Rspo1*-*null* myoblasts expressed similar levels of *Pax7* transcript (Figure S2C) and more than 98% of the cells expressed PAX7 protein (Figure S3B). Immunostainings for the mitosis marker phospho-HIST1H3 (Figure S3D) and the cell-cycle marker MKI67 (Figure S3E) did not reveal any differences in proliferation between *Rspo1 null* and control cells. Likewise, quantification of the proportion of cells engaged in S-phase by 5-ethynyl-2′-deoxyuridine (EdU) incorporation assay (Figure S3F) did not show any difference between control and *Rspo1*-null cells in either high serum (Figure S3G) or low serum (Figure S3H) culture conditions. Our results demonstrate that *Rspo1* is not required for myogenic progenitor expansion from MuSCs.

### *Rspo1* Positively Controls Muscle Cell Differentiation through the Canonical Wnt/β-Catenin Pathway

**Figure 3:**
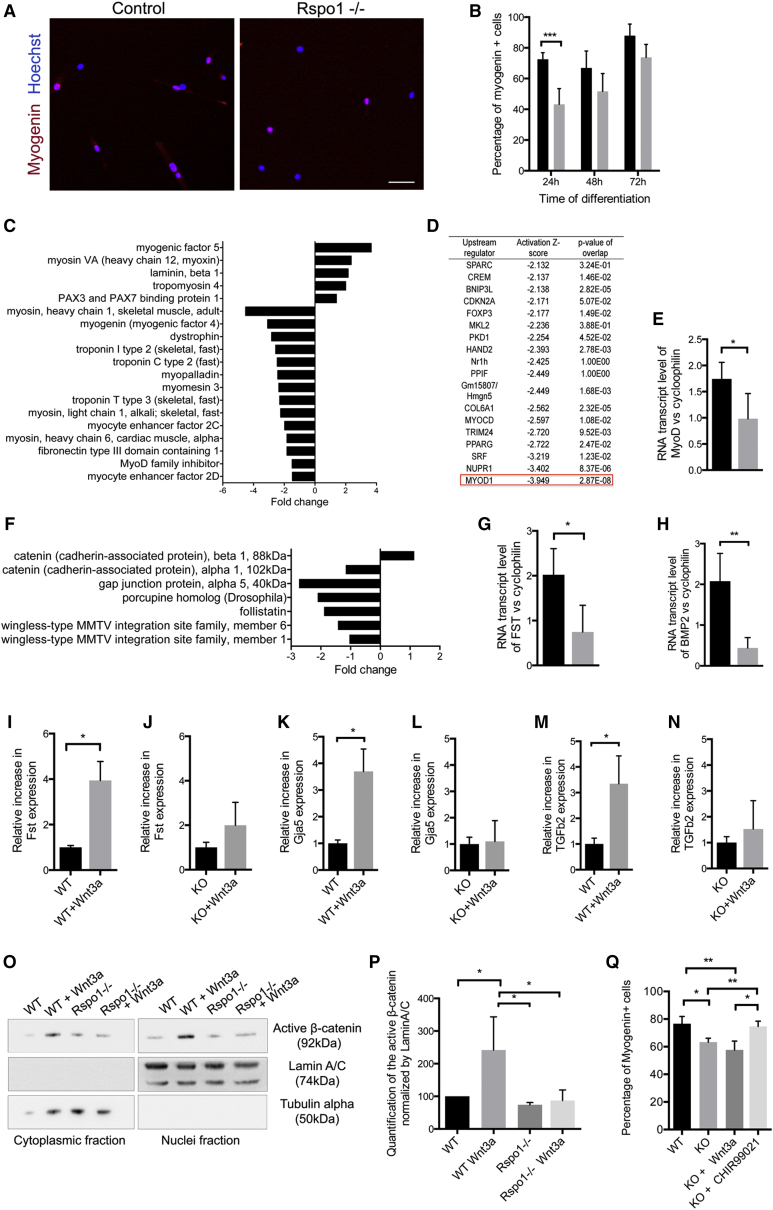
*Rspo1-null* Cells Have a Differentiation Defect Due to an Impaired Activation of the Wnt/β-Catenin Pathway (A) MYOGENIN (red) immunolocalization in control and *Rspo1-null* myocytes. Scale bar, 50 μm. (B) Quantification of differentiated cells. (C) List of genes involved in muscle development and regulated by Rspo1 depending on their fold change in gene expression in *Rspo1-null* versus control myocytes. (D) Upstream Regulators analysis of *Rspo1-null* myocytes transcriptome. (E) qPCR analysis of *MyoD* expression. (F) List of Rspo1-regulated genes involved in canonical Wnt pathway. (G and H) qPCR analysis of *Fst* (G) and (H) *Bmp2* expression levels in differentiated *Rspo1-null* and control myocytes. (I, K, and M) Increased expression of *Fst* (I), *Gja5* (K), and *Tgf*β*2* (M) following WNT3A treatment in control cells. (J, L, and N) Unchanged expression of *Fst* (J), *Gja5* (L), and *Tgf*β*2* (N) following WNT3A treatment in *Rspo1-null* cells. (O and P) Western blot (O) and quantification (P) of active β-CATENIN showing a decrease in β-CATENIN protein levels in nuclei of *Rspo1-null* primary myoblasts treated with WNT3A. (Q) Quantification of differentiated cells after a WNT3A- or a CHIR99021-treatment, demonstrating that the forced-activation of the Wnt/β-catenin pathway in *Rspo1-null* cells restores a control state of differentiation. Nuclei are stained with Hoechst (blue). Error bars indicate SD. ^∗^p value < 0.05; ^∗∗^p value < 0.01; ^∗∗∗^p value < 0.001.

We further induced *Rspo1-null* and control primary myoblasts to differentiate in vitro and quantified the number of myocytes expressing MYOGENIN at different time points (Figure 3A). Similar to our in vivo observations, *Rspo1*-*null* myocytes showed a significant differentiation delay compensated after 2 days in differentiation medium (Figure 3B). To understand the molecular mechanisms regulated by Rspo1 in muscle cells, we analyzed gene expression of *Rspo1*-*null* and control myocytes using Affymetrix microarrays. The expression data identified downregulation of a large number of genes implicated in skeletal myogenesis in *Rspo1*-*null* cells (Figure 3C). The Ingenuity Pathway Analysis (IPA) software identified MyoD as the most prominent downregulated upstream regulator (Figure 3D). We further validated the downregulation of *MyoD* expression by qRT-PCR (Figure 3E).

The transcriptome analysis further highlighted a downregulation in the expression of a set of genes implicated in canonical Wnt pathway in *Rspo1-null* cells (Figure 3F). We validated reduced expression levels of the β-catenin target genes *Fst* (Figure 3G) and *Bmp2* (Figure 3H) by qRT-PCR. We then investigated if Wnt/β-catenin target genes expressions could be induced in *Rspo1*-*null* cells following exogenous ligand stimulation. Following WNT3A protein treatment, control myocytes exhibited elevated expression of the β-catenin responsive genes *Fst*, *Gja5*, and *Tgfb2* (Figures 3I, 3K, and 3M) (Rudolf et al., 2016). In contrast, *Rspo1*-*null* myocytes did not respond to exogenous WNT3A stimulation (Figures 3J, 3L, and 3N). Quantification of active-β-CATENIN proteins in nuclear and cytoplasmic fractions (Figure 3O) showed that β-CATENIN was de-phosphorylated and translocated to the nuclei following WNT3A stimulation only in control cells (Figure 3P). Because activation of Wnt/β-catenin signaling is required in muscle progenitor cells to support differentiation (Rudolf et al., 2016), we hypothesized that a lack of β-CATENIN activation in *Rspo1-null* myocytes could explain the observed delay in differentiation. To stabilize β-CATENIN proteins in muscle cells, independently of the extracellular ligands, we used the small molecule CHIR99021 to inhibit GSK3 and subsequently the β-catenin destruction complex. Quantification of the number of differentiated cells showed that CHIR99021-mediated activation of the Wnt/β-catenin pathway, which acts downstream of the receptor, restored a control state of differentiation in *Rspo1-null* cells (Figure 3Q). Moreover, treatment of *Rspo1-null* myoblasts with CHIR99021 rescued the expression of the β-catenin target genes (Figure S4). Taken together, our data suggest that RSPO1 is required for the activation of the canonical Wnt pathway during myogenic differentiation.

### *Rspo1* Limits Cell Fusion

**Figure 4:**
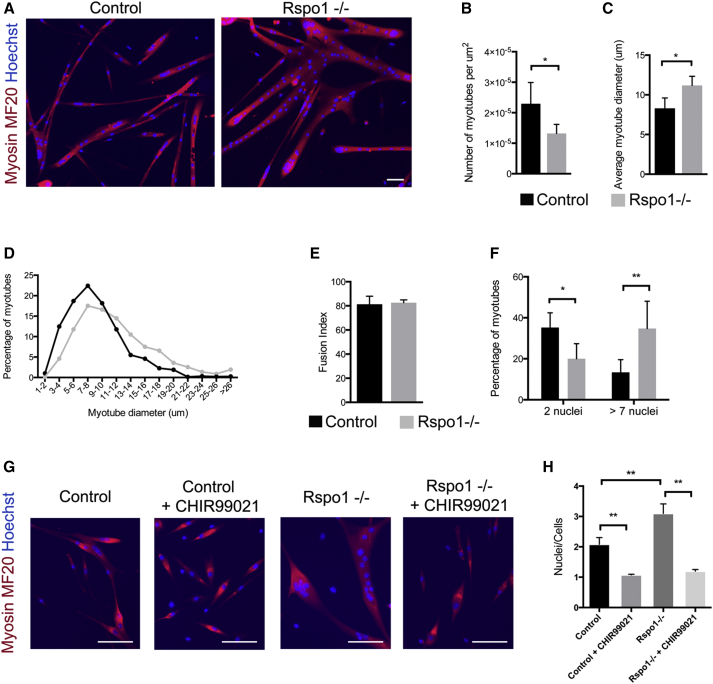
*Rspo1-null* Cells Show Enhanced Fusion (A) MYHC immunolocalization (red) of myotubes after 4 days of differentiation. (B) Quantification of the number of myotubes per surface. (C) Mean diameter of myotubes showing that *Rspo1-null* myotubes are 30% bigger than control myotubes. (D) Distribution of the percentage of myotubes depending on their diameter. (E) Quantification of the number of fused cells normalized to the total number of cells. (F) Quantification of the number of nuclei per myotube. (G) Myocytes were treated with CHIR099021 24 hr after induction of differentiation. MYHC immunolocalization (red) in myotubes after 4 days of differentiation. (H) Quantification of the number of nuclei per cell. Activation of canonical Wnt signaling by CHIR99021 completely blocks muscle cell fusion. Nuclei are stained with Hoechst (blue). Scale bars, 50 μm. Error bars indicate SD. ^∗^p value < 0.05; ^∗∗^p value < 0.01; ^∗∗∗^p value < 0.00.

We next investigated muscle cell fusion in *Rspo1-null* and control primary myocytes. After 4 days in differentiation media, control myocytes fused and generated both small and large myotubes, while *Rspo1*-*null* cells generated much larger syncytia (Figure 4A). Consequently, the number of myotubes per surface was reduced in *Rspo1*-*null* cultures (Figure 4B), and the average diameter of *Rspo1-null* myotubes was higher compared to control myotubes (Figure 4C). Graphical distribution of myotube size further demonstrated that this increase was homogeneous and not due to the appearance of a specific sub-population (Figure 4D). Importantly, while the percentage of nuclei that underwent fusion was similar in cultures of both genotypes (Figure 4E), we observed a significantly higher proportion of myotubes containing more than seven nuclei in *Rspo1*-*null* cultures (Figure 4F). We next treated myocytes after 24 hr of differentiation with CHIR99021 and let them fuse for 2 more days in vitro. CHIR99021 treatment completely abolished muscle cell fusion of both control and *Rspo1-null* cells (Figures 4G and 4H). These observations indicate that even though canonical Wnt signaling is required for proper commitment to the differentiated state, it is inhibitory for myocyte fusion. Our results indicate that *Rspo1* negatively regulates muscle cell fusion and that its absence leads to the generation of larger myotubes containing more nuclei.

### *Rspo1* Negatively Regulates the Non-canonical Wnt7a/Fzd7 Pathway

Our data suggest that Rspo1 has contradictory roles in muscle progenitor cell differentiation and fusion. We thus hypothesized that *Rspo1* depletion could also impact the non-canonical Wnt pathway. It is known that WNT7A stimulates the migration of MuSCs and primary myoblasts through RAC1 activation (Bentzinger et al., 2014). We thus validated that WNT7A stimulation of wild-type differentiating myocytes results in enhancement of muscle fusion, as shown by increased myotube size (Figure S5A) and a higher number of myonuclei (Figures S5B and S5C). As such, upregulation of non-canonical Wnt7a signaling could explain the increased fusion we observed in *Rspo1-null* muscle cells.

**Figure 5:**
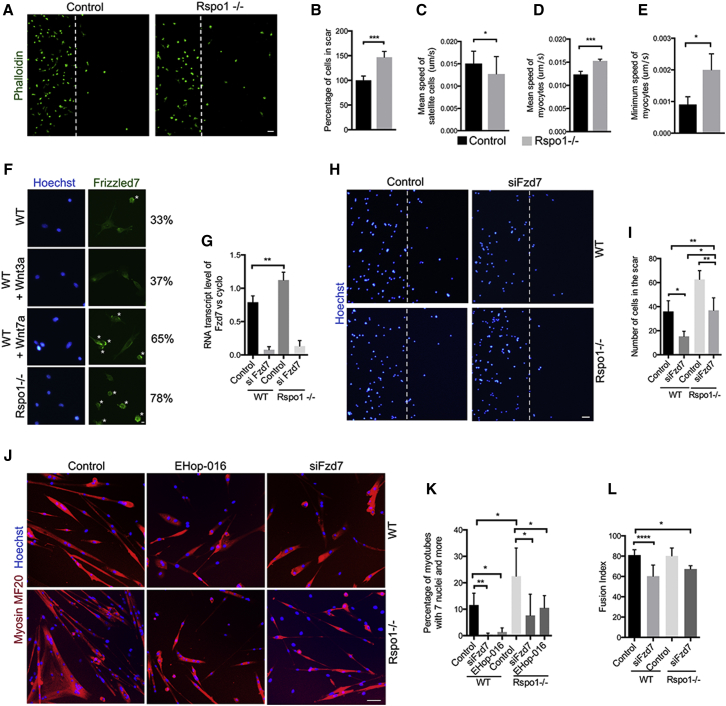
*Rspo1* Depletion Potentiates Wnt7a/Fzd7/Rac1 Signaling (A) Phalloidin (green) staining of myocytes 24 hr after scratching confluent-state cells. White bars show the limits of the scratch. (B) Quantification of the number of cells in the scar 24 hr after the scratch showing increases migration of *Rspo1-null* myocytes. (C) Quantification of the mean speed of MuSCs on single myofiber. (D and E) Quantification of the mean speed (D) and of the minimum speed (E) of myocytes. (F) FZD7 (green) immunolocalization in myocytes treated by WNT3A and WNT7A recombinant proteins. Asterisks represent FZD7 proteins located at the cell membrane. Percentages of cells with polarized FZD7 staining are indicated on the right. (G) qPCR analysis of *Fzd7* expression in myocytes confirming siRNA efficiency. (H) Myocytes 24 hr after scratching confluent-state cells upon siFzd7 treatment. White bars show the limits of the scratch. (I) Quantification of the number of migrating cells shows a decreased migration upon *Fzd7* silencing. (J) MYHC (red) immunolocalization in myotubes treated with siFzd7 or EHop16. (K) Quantification of the number of myotubes with more than seven nuclei. (L) Fusion index of myotubes upon siFzd7 treatment. Nuclei are stained with Hoechst (blue). Scale bars, 50 μm (A, H, and J); 20 μm (F). ^∗^p value < 0.05; ^∗∗^p value < 0.01; ^∗∗∗^p value < 0.001; ^∗∗∗∗^p value < 0.0001. Error bars correspond to SD.

To test this hypothesis, we performed scratch-wound assays (Figure 5A) and observed that *Rspo1* deficiency increased myocyte migration (Figure 5B). Time-lapse imaging on single myofibers (Otto et al., 2011) showed that *Rspo1-null* MuSCs has lower velocity than control cells (Figure 5C). Interestingly, time-lapse imaging of myocytes in wound assays revealed that *Rspo1* deficiency significantly increased both the mean velocity (Figure 5D) and the minimum speed (Figure 5E) of migrating cells. These results indicate that *Rspo1* inhibits the motility of myogenic progenitor cells, but not MuSCs, during directed migration.

The major receptor of WNT7A in myogenic cells is FRIZZLED7 (FZD7) (Le Grand et al., 2009) that accumulates at the periphery of moving cells during migration (Bentzinger et al., 2014). Immunolocalization of FZD7 in primary myocytes demonstrated that while few cells show FZD7 accumulation in control and WNT3A-treated conditions, FZD7 staining appeared polarized in both WNT7A-treated cells and *Rspo1-null* cells (2-fold increase) (Figure 5F). We also observed a slight increase in *Fzd7* gene expression in *Rspo1*-*null* cells (Figure 5G). Altogether, these results suggest an enhancement of non-canonical Wnt7a/Fzd7 signaling in *Rspo1-null* cells.

We next transfected both control and *Rspo1*-*null* cells with small interfering (siRNA) directed against *Fzd7* (Figure 5G). We quantified the number of migrating cells in scratch wound assays (Figure 5H) and observed that siFzd7 treatment decreased cell migration in both control and *Rspo1 null* cells. Importantly, *Rspo1*-*null* cells treated with siFzd7 migrated at a rate similar to control cells, indicating that the increase in cell motility observed in *Rspo1*-*null* cells was related to an increase in Wnt7a/Fzd7 signaling (Figure 5I). We then differentiated control and *Rspo1*-*null* myocytes in control and *Fzd7* silencing conditions or in the presence of RAC1 inhibitor EHop-016 (Montalvo-Ortiz et al., 2012) (Figure 5J). Strikingly, treatment of *Rspo1*-null cells with either siFzd7 or EHop-16 restored the proportion of large myotubes containing more than seven nuclei to a percentage similar to untreated control cells (Figure 5K). Interestingly, siFzd7 treatment resulted in a small reduction in fusion index in both controls and *Rspo1-null* cells (Figure 5L).

In summary, our results demonstrate that *Rspo1* negatively regulates muscle cell migration and fusion by dampening non-canonical Wnt7a/Fzd7/Rac1 pathway. Our data suggest that, in MuSC progeny, *Rspo1* integrates antagonistic Wnt pathways for fine-tuning of muscle architecture during tissue repair.

## Discussion

In most mammalian tissues, the R-spondin family of secreted proteins positively regulates the canonical Wnt signaling pathway. Canonical Wnt signaling plays an important role in adult muscle regeneration by allowing for the timely differentiation of MuSC descendants during tissue repair (Figeac and Zammit, 2015, Rudolf et al., 2016). Here, we show that the absence of *Rspo1* expression significantly affects myogenic progenitor cell differentiation both in vitro and in vivo and is associated with a defect in canonical Wnt/β-catenin activation. These data are consistent with previous reports demonstrating that RSPO1 is a regulator of tissue-resident stem cells such as the mammary gland (Cai et al., 2014), the skin (Li et al., 2016), or the intestine (de Lau et al., 2011).

Our results show that *Rspo1* is dispensable for muscle development. This is also the case for other members of the family, and this outcome could be explained through compensation. In contrast, we show that *Rspo1* is essential for adult muscle regeneration. Following injury, *Rspo1-null* muscle progenitor cells show a transient delay in differentiation, but *Rspo1-null* muscles go on to regenerate efficiently and are even composed of bigger myofibers containing more myonuclei. This increase in fusion is due to a concomitant downregulation of canonical Wnt/β-catenin signaling and upregulation of non-canonical Wnt7a/Fzd7/Rac1 signaling pathways in muscle progenitor cells. Therefore, *Rspo1-null* muscle cells migrated faster and fused with a higher efficiency compared to wild-type cells, and these phenotypes were normalized by *Fzd7* silencing or RAC1 inhibition. Importantly, while it has been shown that RSPO3 can activate the non-canonical Wnt pathway during *Xenopus* embryonic development (Ohkawara et al., 2011), the impact of R-spondins on non-canonical Wnt pathways has not been previously demonstrated in adult mammalian cells. Further work would be to investigate the role of R-spondin proteins in controlling non-canonical Wnt pathways in other adult stem cells.

Activation of the Wnt7a/PCP pathway in MuSCs induces their symmetrical expansion. As such, Wnt7a overexpression enhances muscle regeneration and increases MuSC numbers (Le Grand et al., 2009). In contrast, while we observed that *Rspo1* deficiency resulted in over-activation of Wnt7a/Fzd7/Rac1 pathway in differentiating myocytes, we did not find any differences in the number of Pax7-expressing myogenic progenitors during muscle regeneration or in the MuSCs pool after regeneration is finished. Our data suggest that RSPO1 does not influence the Wnt/PCP pathway in MuSC. We hypothesize that this stage-specific requirement for RSPO1 action is related to the fact that canonical Wnt/β-catenin signaling is inactive in MuSC, but activated in differentiating myogenic progenitor cells (Rudolf et al., 2016). We propose that *Rspo1* controls the antagonistic balance between canonical and non-canonical Wnt pathways specifically in differentiating muscle cells.

Both canonical and non-canonical Wnt signaling induce distinct cellular and molecular processes but share several core components. Thus, the exclusive activation of a Wnt pathway is possible by the selective interaction between specific ligands and receptors. More precisely, it has been shown that WNT5A can inhibit or activate β-catenin signaling depending on the presence of specific Frizzled receptors at the membrane (Mikels and Nusse, 2006). It is also well documented that canonical and non-canonical Wnt ligands can compete for the activation of their selective pathways. Non-canonical WNT5A can antagonize Wnt/β-catenin activation in *Xenopus* (Larabell et al., 1997) and in mammalian hematopoietic stem cells (Nemeth et al., 2007). The increase in non-canonical Wnt signaling observed in *Rspo1-null* muscle cells could then be related to a change in the composition of surface receptors. We propose that Rspo1 has a role in maintaining the “canonical” Frizzled receptors at the surface of muscle cells, and its absence can lead to a preferential increase in the availability of non-canonical Frizzled receptors. The increase in FZD7 membrane expression in *Rspo1-null* cells may start answering this question, but further work will be dedicated to exploring the expression pattern of the different Frizzled receptors expressed at the surface of MuSCs and their descendants, their dynamics during myogenic commitment, and myotube formation.

*Rspo1*-deficient muscles can regenerate efficiently and have an increase in nuclear content. The generation of larger myofibers containing fewer myonuclei has been described as pathological because it can perturb muscle homeostasis and function. An example is the hyper-muscular *Myostatin null* mice, in which fiber enlargement is not accompanied by a commensurate increase in nuclear content and is postulated to lead to compromised physiological performance in terms of force generation and fatigability (Amthor et al., 2007, Mouisel et al., 2014). A number of studies have suggested that there is a finite nuclear to cytoplasmic volume that supports normal cellular function that when exceeded, as in the case of *Myostatin null* muscle, leads to functional impairment (Matsakas et al., 2013). Interestingly, our analysis of the contractile properties of regenerated *Rspo1*-null muscles indicated that, while containing more nuclei, the regenerated myofibers were functional. We thus suggest that RSPO1 protein could serve as a therapeutic treatment in muscle pathologies that show aberrant or elevated muscle progenitor fusion.

## Experimental Procedures

### Mice

Experimental animal protocols were performed in accordance with the guidelines of the French Veterinary Department and approved by the University Paris-Descartes Ethical Committee for Animal Experimentation. All experiments were performed in 6- to 9-week-old mice. Our animals are on C57B6N/SV129 genetic background. *Rspo1-null* mice genotyping has been described previously (Chassot et al., 2008).

### Muscle Stem Cells Isolation and Primary Myoblasts Culture

Skeletal muscles of mice were dissected (quadriceps, TA, EDL, gastrocnemius, soleus, gluteus) and transferred to a sterile Petri dish on ice. Muscles were incubated in a solution of 1.5 U/mL of collagenase B, 2.4 U/mL of DispaseII, and 2 M of CaCl_2_ for 45 min at 37°C with periodic mechanical digestion. Fetal bovine serum was added to stop the digestion. After centrifugation, pellet was resuspended in growth medium consisting of Ham’s F10 (Life Technologies) with 20% FBS (Eurobio), 1% Pen/Strep (Life Technologies), and 2.5 ng/μL basicFGF (R&D Systems). Satellite cells were then purified using MACS cell separation system, according to the manufacturer’s protocol. Satellite cells were then allowed to proliferate and give rise to primary myoblasts after two passages. If needed, primary myoblasts were differentiated using a differentiation medium composed of DMEM (GIBCO) with 2% horse serum (GIBCO), and 1% Pen/Strep (GIBCO). Cells were treated with 50 ng/mL of recombinant WNT3A and/or WNT3A proteins (R&D Systems) for a minimum of 24 hr or with the RAC1 inhibitor eHop-016 (Sigma) at 1.5 μM for 8 hr.

### Immunostaining and EdU Incorporation on Primary Myoblasts

Primary myoblasts were fixed for 8 min with 4% PFA in PBS then incubated with 0.2% Triton in PBS for 20 min at room temperature. Cells were incubated with primary antibodies during 1 hr at room temperature, followed by PBS washes and a 1 hr incubation with the secondary antibodies. Nuclei were stained with Hoechst. Primary antibodies used were R-spondin1 (Sigma), Phospho-HIST1H3 (Cell Signaling), Pax7 (Santa Cruz), Myogenin (Santa Cruz), M-Cadherin (BD Biosciences), MYHC (R&D Systems), and MKI67 (Santa Cruz). EdU incorporation was performed with Click-iT EdU Imaging Kits from Invitrogen, according to the manufacturer’s protocol. Cells were incubated with EdU for 1 hr at 37°C.

### Scratch Assay

Cells were treated with 10 μg/mL of Mitomycin-C (Sigma) for 8 hr before scratching the monolayer of cells in a straight line. Plates were washed few times with PBS and primary myoblasts were incubated in growth medium for 24 hr at 37°C. If needed, WNT7A recombinant proteins (100 ng/mL from R&D Systems) were added in the culture medium. Cells were then fixed with 4% PFA-PBS, and nuclei were stained with Hoechst. The numbers of nuclei in the scar were counted manually.

### RNA Interference

Primary cells were seeded in collagen-coated plate in growth medium without Pen/Strep. siRNA transfection was performed using Lipofectamine 2000 and OptiMEM according to the manufacturer’s protocol. Both siFzd7 and siControl (Ambion) were used at a 50-μM concentration.

### Live Imaging

Cell imaging was performed on a microscope (Zeiss Axio Observer.Z1) with 10× objective under 5% CO_2_ at 37°C in differentiation media. Image acquisition was performed with Metamorph software (Molecular Devices) with time points acquired every 8 min over 24 hr. Velocity was calculated with ImageJ software. The minimum velocity is defined as the lowest velocity obtained by cells during the tracking period.

### qPCR

RNA was isolated either by RNeasy Mini Plus Kit (QIAGEN) or by TRIzol Reagent followed by a DNase treatment (Life Technologies). The reverse transcription was performed with 100 ng of RNA using the high-capacity cDNA Reverse Transcription kit (Applied Biosystems). Transcript levels were determined by LightCycler 480 Real-Time PCR System (Roche) using SYBR green I Master (Roche). The melting curves were checked for each experiment, and the primers efficiency was calculated by serial sample dilution. Targeted gene expressions were normalized by cyclophilin reference gene. Sequences of primers used are given in the Supplemental Experimental Procedures.

### Image Acquisition and Quantitative Analysis

Image acquisitions were performed at the Cochin Institute and the ICM Imaging Facilities in Paris. Immunofluorescent stainings were analyzed with an Olympus BX63F microscope, Zeiss Axio Observer.Z1 microscope and AZ100 Nikon Macroscope from Cochin Institute, and Zeiss Axiovert 200M microscope and Axio Scan.Z1 microscope from the ICM. We also performed acquisition on EVOS FL Cell Imaging System microscope. Fluorochromes Alexa Fluor 488 and Alexa Fluor 546 (Life Technologies) were used. NIS-Element (Nikon), Metamorph (Molecular Devices), and Photoshop software (Adobe) were used for image acquisition. Quantifications were performed with ImageJ software.

### Statistical Analysis

Analysis were performed using three to five mice and three to six biological replicates for cell experiments. Statistical analysis were done using GraphPad software (Prism) with unpaired two-tailed Student’s t test, the one-way ANOVA, or the two-way ANOVA. p < 0.05 was considered statistically significant. Data were expressed as mean ± SD.

## Author Contributions

Conceptualization, F.L.G. and A.-A.C.; Methodology, C.F.B., A.F., K.P., M.A.R., and M.-C.C.; Investigation, F.L., E.V., M.-C.S., R.M., L.G., and A.-A.C.; Writing – Original Draft, F.L. and F.L.G.; Writing – Review & Editing, F.L., M.-C.S., C.F.B., K.P., M.-C.C., A.-A.C., and F.L.G.; Supervision, K.P., M.A.R., M.-C.C., and F.L.G.; Funding Acquisition, F.L.G.; Project Administration, F.L.G.

## Accession Numbers

The accession number for the microarray data reported in this paper is GEO: GSE84016.

## Supplemental Information

Document S1. Supplemental Experimental Procedures, Figures S1–S5, and Table S1

Document S2. Article plus Supplemental Information
