# Supplementary material for: R-spondin1 Controls Muscle Cell Fusion through Dual Regulation of Antagonistic Wnt Signaling Pathways
Source: Cell Rep. 2017 Mar 7;18(10):2320–30. doi: 10.1016/j.celrep.2017.02.036 (PMC5357729; doi:10.1016/j.celrep.2017.02.036)
Supplement: Document S1. Supplemental Experimental Procedures, Figures S1–S5, and Table S1 [file mmc1.pdf]

Cell Reports, Volume *18*

Supplemental Information

R-spondin1 Controls Muscle Cell Fusion through Dual Regulation of Antagonistic Wnt Signaling Pathways

Floriane Lacour, Elsa Vezin, C. Florian Bentzinger, Marie-Claude Sincennes, Lorenzo Giordani, Arnaud Ferry, Robert Mitchell, Ketan Patel, Michael A. Rudnicki, Marie-Christine Chaboissier, Anne-Amandine Chassot, and Fabien Le Grand

SUPPLEMENTARY FIGURES

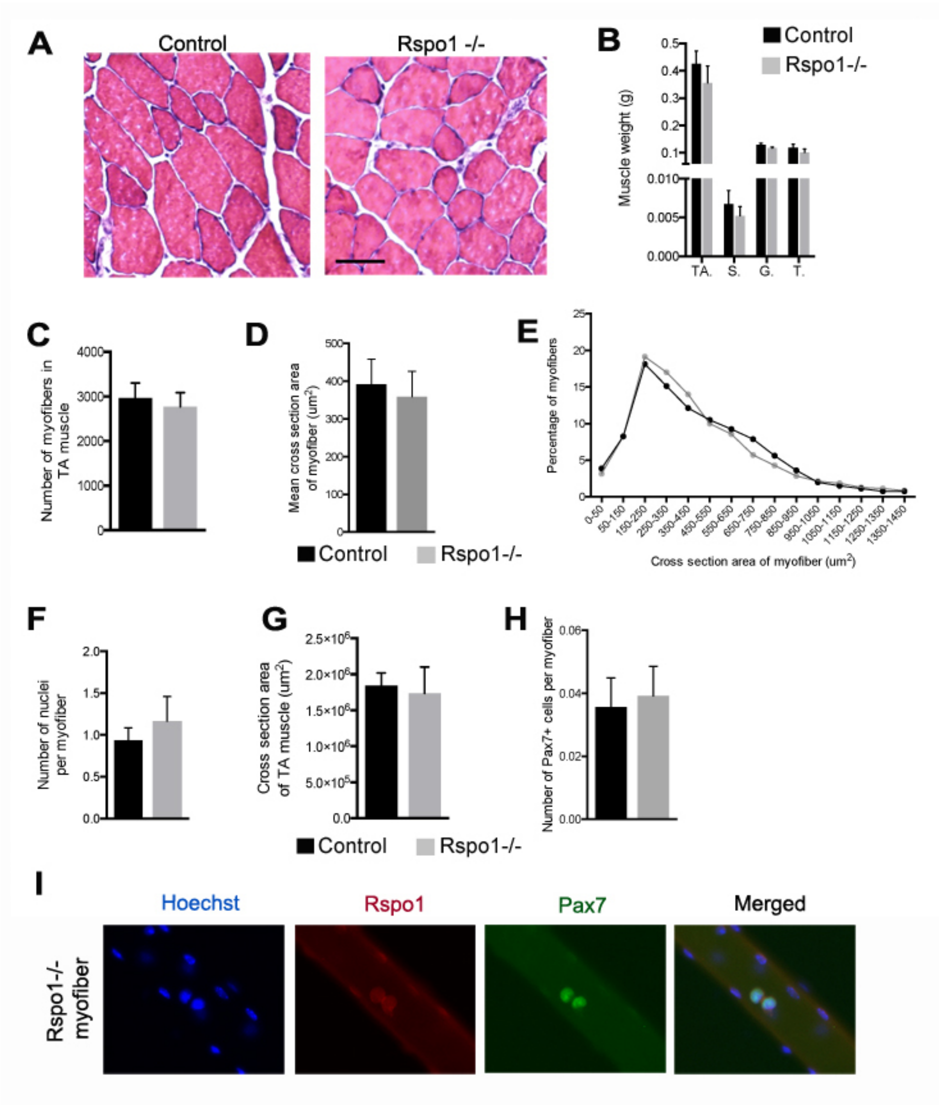

Figure S1. ***Rspo1* gene deletion does not alter muscle tissue structure. Related to Figure 2.**
(A) Hematoxylin and Eosin staining in uninjured TA cryosections. (B) Quantification of the Tibialis Anterior (TA), Soleus (S), Gastrocnemius (G) and Triceps (T) muscle weight showing no difference between control and *Rspo1-null* mice. (C) Quantification of the total number of myofiber in Tibialis Anterior muscle showing no difference between control and mutant condition. (D) Mean cross section area of myofibers and (E) distribution of the number of myofibers depending on their area in control and mutant TA muscle. (F) Quantification of the number of nuclei per myofiber. (H) Mean cross section area of the Tibialis Anterior muscle. (I) Quantification of the number of Pax7-positive cells per myofiber. (I) Immunolocalization of RSPO1 (red) and PAX7 (green) proteins in MuSCs on cultured single myofibers from Rspo1-null animals. Nuclei are stained with Hoechst (blue).
Error bars indicate standard deviation. Insert panels show a 2,2 X enlargement.

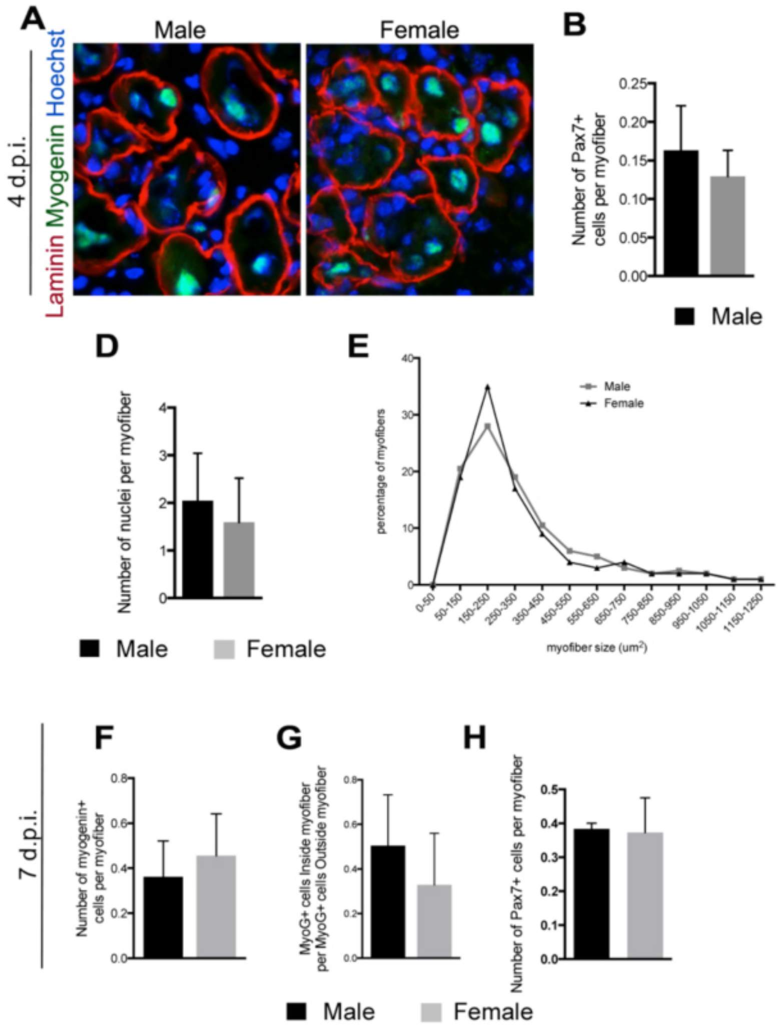

Figure S2. **No differences in muscle regeneration were observed between male and female *Rspo1-null* mice. Related to Figure 2.**
Data of *Rspo1-null* samples from figures 2A to H are shown here expressed as male or female. No differences were observed between muscle tissues of either gender.

(A) Immunolocalization of LAMININ (red) and MYOGENIN (green) proteins in *Rspo1-null* TA muscles at 4d.p.i. Nuclei are stained with Hoechst (blue). (B) Quantification of the number of PAX7-positives cells per myofiber 4 d.p.i. (C) Quantification of the number of cells expressing MYOGENIN per myofiber at 4 d.p.i. (D) Quantification of the number of nuclei per myofiber at 4 d.p.i. (E) Distribution of cross sectional area of myofibers in TA muscles at 7d.p.i. (F) Quantification of the number of cells expressing MYOGENIN per myofiber at 7 d.p.i. (G) Quantification of the number of MYOGENIN-positives cells inside the myofibers normalized by the number of cells outside the myofibers. (H) Quantification of the number of PAX7-positives cells per myofiber at 7 d.p.i.

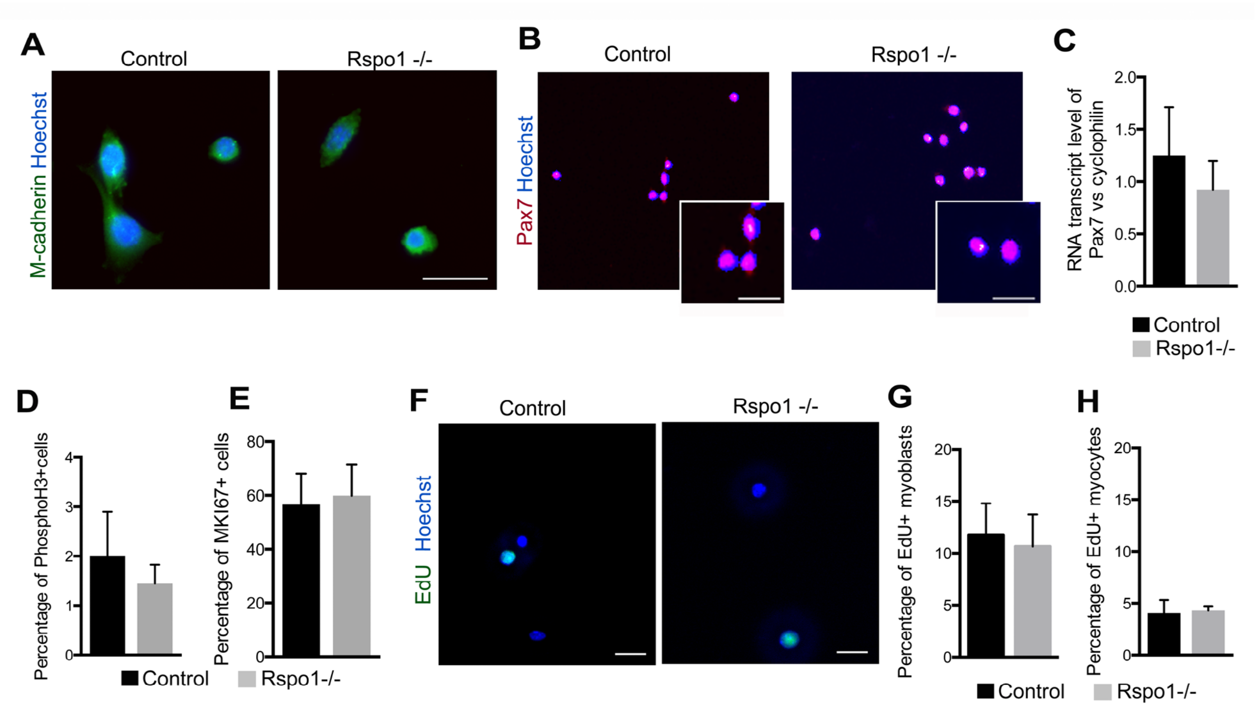

Figure S3. ***Rspo1-null* cells proliferate properly. Related to Figure 3.**
(A) Immunolocalization of M-cadherin proteins in control and *Rspo1-null* myoblasts. Nuclei are stained with Hoechst (blue). Bars : 50 µm. (B) Immunolocalization of Pax7 proteins in control and *Rspo1-null* myoblasts. Nuclei are stained with Hoechst (blue). Bars : 30 µm. (C) Gene expression of Pax7 in myoblasts by qPCR, showing that a depletion of Rspo1 does not change Pax7 gene expression. (D) Quantification of the percentage of cells engaged in mitotic phase, by Phospho-Histone H3 staining. (E) Quantification of the percentage of proliferating cells, by KI67 staining, showing no differences of cell number engaged in G1, S, G2 and M phases. (F) Immunostaining of EdU incorporation in control and Rspo1$^{-/-}$ proliferating myoblasts after 1h EdU treatment. Bars : 20 µm. (G) Quantification of the number of proliferating cells and (H) differentiating cells with EdU incorporation.
Error bars correspond to standard deviation. Inset panels show a 2,5 X enlargement.

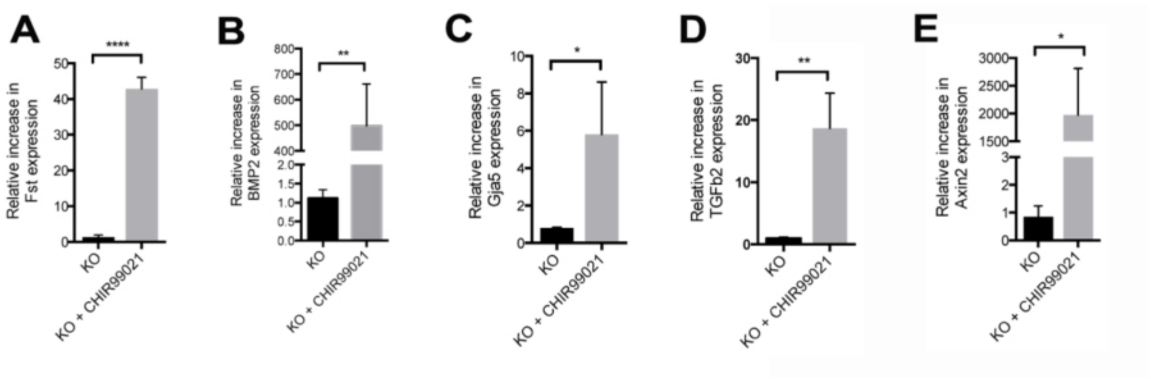

Figure S4. **qPCR analysis of Wnt target genes expression in *Rspo1-null* cells following CHIR99021 treatment. Related to Figure 3.**
Activation of β-catenin by GSK3 inhibition restores the expression of target genes in *Rspo1-null* cells.
*Error bars indicate standard deviation. *: p value <0.05. **: p value <0.01.* **** p value <0.0001

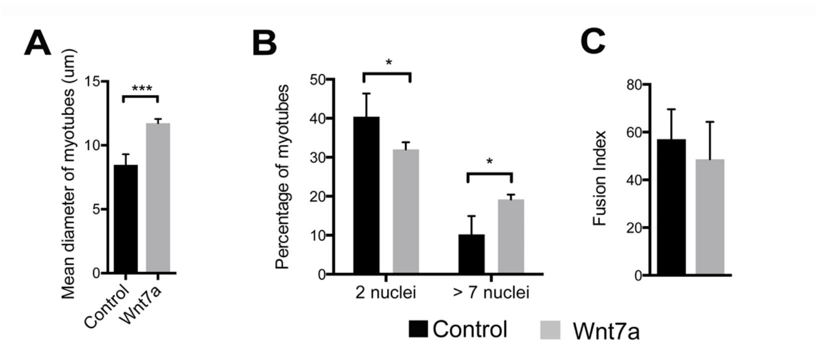

Figure S5. **Wnt7a stimulate muscle fusion. Related to Figure 4.**
(A) Mean diameter size of control and Wnt7a-treated cells, after 4 days of differentiation. (B) Quantification of the number of nuclei per myotubes showing a decrease proportion of Wnt7a-treated-myotubes with 2 nuclei and an increase number of Wnt7a-treated-myotubes with a high number of nuclei. (C) Quantification of the percentage of nuclei in fused cells normalized by the total number of nuclei.
*Error bars indicate standard deviation. *: p value <0.05.* *** p value <0.001

SUPPLEMENTARY METHODS

Cardiotoxin injury

Mice were anaesthetized by intraperitoneal injection of Ketamin at 0,1mg per gram body weight and Xylazin at 0,01mg per gram body weight diluted in saline solution. After having cleaned the mouse hind legs with alcohol, TA muscles were injected with 50µl of cardiotoxin solution (Latoxan, 12 µM in saline) using an insulin needle (Vignaud et al., 2007).

Muscle histology and immunohistochemistry

For cryosections, TA were embedded in Tissue-Tek O.C.T. compound from Cell Path. Muscles were then frozen on the cooled isopentane and cut at a thickness of 10 µm with a Leica cryostat. Frozen muscle were stored at -80°C. Sections were fixed with 4% PFA in PBS for 20 min and permeabilized with cooled-methanol. Antigen retrieval was performed with the Antigen Unmasking Solution (Vector) at 95°C for 10 min. Sections were then blocked with 4% BSA, 5% Goat serum in PBS during 3h and incubated with primary antibody overnight at 4°C. Alexa Fluor secondary antibody was incubated on sections 1h at room temperature and nuclei were stained with Hoechst. Slides were mounted in fluorescent mounting medium from Dako. Primary antibodies used were Laminin (Santa Cruz), Pax7 (Santa Cruz), Myogenin (Santa Cruz) and Dystrophin (Leica). For Hematoxilin/Eosin staining, sections were incubated in Hematoxilin bath for 4 min and with Eosin for 2 min and thereafter dehydrated in 70%, 90% and 100% ethanol and fixed in Xylene bath for 2 min.

Chromatin immunoprecipitation

Primary myoblasts overexpressing full-length Pax7 (cloned into the pBrit plasmid vector) were generated by retroviral infection and cultured as previously described (McKinnell et al., 2008). Primary myoblasts were cross-linked using 1% formaldehyde in PBS 1X for 10 minutes. Glycine was then added to a final concentration of 0,125M for 5 minutes, followed by centrifugation. Cell pellet was washed in PBS 1X, and resuspended in ChIP lysis buffer (50mM Tris-HCl pH 8.0, 10mM EDTA, 0,5% SDS). Chromatin was fragmented using Covaris sonicator. Before ChIP, equal volume of ChIP assay buffer (20mM Tris-HCl pH 8.0, 1,2mM EDTA, 1,1% Triton X-100, 200mM NaCl) was added to adapt the concentration of salts and detergents. Immunoprecipitation was performed using 1mg of chromatin and 30 µl of anti-Flag M2 affinity gel (Sigma) for 3h at 4 °C. Antibody–protein–DNA complexes were collected, washed and eluted, and cross links were reversed according to manufacturer's instructions. DNA was purified by phenol/chloroform purification using linear acrylamide (Ambion) and GlycoBlue (Ambion) as carriers. ChIP enrichment was analyzed by quantitative PCR using Mx3000P (Stratagene).

Microarray and bioinformatics

The RNA from primary myoblasts were isolated using TRIzol Reagent from Life Technologies according to the manufacturer's protocol. The purity and the quality of the RNA were performed by Bioanalyzer 2100, using Agilent RNA6000 nano chip kit (Agilent Technologies) at the Cochin Institute Genomic facility. RNA were reverse

transcribed using the Ovation PicoSL WTA System (Nugen). The product cDNA were then hybridized to GeneChip Mouse Gene 2.1 ST (Affymetrix). The Affymetrix data were normalized using R software (R Development Core Team, 2011) and gene expression levels were compared using one-way ANOVA. In R-spondin1 deficiency myocytes, the genes were selected with $p<0,05$ significance. The functional analysis of the gene expression levels was interrogated with Ingenuity pathway (Ingenuity Systems, http://www.ingenuity.com).

Muscle contractility

Tibialis anterior muscles were evaluated by the measurement of *in situ* isometric muscle contraction in response to nerve stimulation. Mice were anaesthetized using a pentobarbital solution (ip, 60 mg/kg) and supplemental doses were given as required to maintain deep anesthesia during experiments. Feet were fixed with clamps to a platform and knee were immobilized using stainless steel pins. The distal tendons of muscles were attached to an isometric transducer (Harvard Bioscience) using a silk ligature. The sciatic nerves were proximally crushed and distally stimulated by bipolar silver electrode using supramaximal square wave pulses of 0.1 ms duration. All data provided by the isometric transducer were recorded and analyzed on a microcomputer, using PowerLab system (4SP, AD Instruments). All isometric measurements were made at an initial length L0 (length at which maximal tension was obtained during the tetanus). Responses to tetanic stimulation (pulse frequency from 75 to 143 Hz) were successively recorded. Maximal tetanic force was determined. Muscle masses (m) were measured to calculate specific maximal force. Finally, the fatigue resistance was assessed. The fatigue protocol consists of one continuous contraction (50 Hz for 45s). The time to reach 50% of the initial force was measured. After contractile measurements, mice were sacrificed with an overdose of anesthetic solution.

Western blot analysis

The nuclear and cytoplasmic protein fractions were obtained with NE-PER Nuclear and Cytoplasmic Extraction reagents kit (Thermo Scientific) according to the manufacturer's protocol. Protein amounts were quantified by BCA Protein Assay Kit (Pearce). In each condition, 30µg of proteins was prepared with home-made Laemmli. Samples were runned on NuPAGE 4-12% BIS-TRIS gels (Life Technologies) and transferred on nitrocellulose membranes. After blocking in 5% milk and 0.1% Tween-20 in TBS, membranes were incubated with primary antibodies overnight at 4°C. Secondary antibodies were then incubated on the membrane for 1h at room temperature. Signals were detected using SuperSignalWest Pico Chemiluminescent Substrate (Thermo Scientific) with ImageQuant TL LAS 4000 from (GE Healthcare). The primary antibodies used were as follows: LaminA/C (Cell Signaling), active β-catenin (Cell Signaling) and alpha-Tubulin (Sigma).

SUPPLEMENTARY TABLE

Table S1 : Primers used in this study. Related to Methods.

**Expression Primers**	
Cyclophilin	AAGAAGATCACCATTTCCGACT
	TTACAGGACATTGCGAGC
MyoD	TACCCAAGGTGGAGATCCTG
	CATCATGCCATCAGAGCAGT
Pax7	CTGGATGAGGGCTCAGAT
	GGTTAGCTCCTGCCTGCTTA
Fst	TGACCTGTAATCGGATTTGC
	TGGAATCCCATAGGCATTTT
Gja5	CAACTTCGACCTCACTCTGAGC
	AGCATGCGGAAAATGAACAGG
Bmp2	AGATCTGTACCGCAGGCACT
	CCGTTTTCCCACTCATCTCT
Tgfb2	ATCGTCGGCTTTGATGTCTC
	GCTGGGTGGGAGATGTTAAG
Fzd7	TATCGCCTACAACCAGACCA
	ACACGGGTGCGTACATAGAG
**ChIP Primers**	
Myf5 -57.5 kb	TGTGGCTCTCTCTCCGTATG
	AATACAGACATGCAGGCTTCAC
Myf5 -111 kb	CATCCCACATAATCCAATCAC
	ACACAGATGGATGGGAAAGA
Rspo1 +35 kb (v1)	CATCAGTGTGTCCTTGGATAG
	GAACAGAGCCACTCCATTT
Rspo1 +35 kb (v2)	AACAAATGGAGTGGCTCTG
	GGCCCTAAAGCTAAGAATGG
Ighd TSS	CTGGACAGAGTGTTTCAAAAC
	CTGGCAGGAAGCAGGTCATGT
Gapdh -2 kb	TCCAGTGAGGACGGTATGAT
	CATAAAGATGGGGCAAAATG

SUPPLEMENTARY REFERENCES

McKinnell, I.W., Ishibashi, J., Le Grand, F., Punch, V.G.J., Addicks, G.C., Greenblatt, J.F., Dilworth, F.J., and Rudnicki, M.A. (2008). Pax7 activates myogenic genes by recruitment of a histone methyltransferase complex. Nat. Cell Biol. *10*, 77–84.

Vignaud, A., Hourdé, C., Butler-Browne, G., and Ferry, A. (2007). Differential recovery of neuromuscular function after nerve/muscle injury induced by crude venom from Notechis scutatus, cardiotoxin from Naja atra and bupivacaine treatments in mice. Neurosci. Res. *58*, 317–323.
